# Tuning Ion Current Rectifying Nanopipettes for Sensitive Detection of Methicillin-Resistant *Staphylococcus aureus*

**DOI:** 10.1021/acs.analchem.4c03510

**Published:** 2025-01-21

**Authors:** Shekemi Denuga, Pallavi Dutta, Dominik Duleba, Guerrino Macori, Séamus Fanning, Robert P. Johnson

**Affiliations:** † School of Chemistry, University College Dublin, Belfield, Dublin D04 N2E5, Ireland; ‡ School of Biology & Environmental & Biological Sciences, University College Dublin, Belfield, Dublin D04 N2E5, Ireland; § UCD-Centre for Food Safety, University College Dublin, Belfield, Dublin D04 N2E5, Ireland; ∥ School of Public Health, Physiotherapy & Sports Science, University College Dublin, Belfield, Dublin D04 N2E5, Ireland

## Abstract

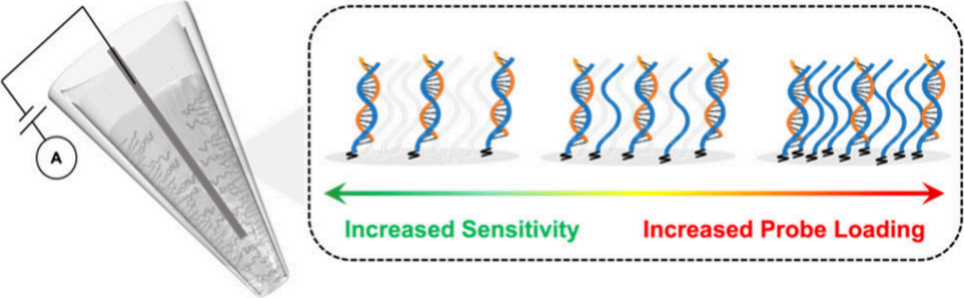

Infectious diseases pose a growing challenge in healthcare, with the increasing rate of antimicrobial resistance limiting therapeutic options available for treatment. Rapid detection of infections at the earliest opportunity can significantly improve patient outcomes. In this report, ion current rectifying quartz nanopipettes with ca. 109 nm orifices were utilized for the label-free detection of DNA indicative of methicillin-resistant *Staphylococcus aureus* (MRSA). By immobilizing probe DNA complementary to the *mecA* gene on the internal walls of the nanopipette, the detection was achieved by monitoring changes in ion current rectification (ICR) following probe–target hybridization. We demonstrate enhanced sensitivity by controlling the surface probe density, resulting in a tunable, sensitive sensor technology with a detection limit as low as 0.35 pM. Finite element simulations are used to support our experimental findings, revealing that to maximize target-induced changes to ICR, the probe surface density must be minimized. This sensitive and label-free methodology was integrated with the polymerase chain amplification reaction to achieve selective identification of this pathogen from laboratory-grown cultures, highlighting that ion current rectifying nanopipette sensors offer the potential to be a cost-effective and rapid tools for infectious disease detection.

In recent years, solid-state conical nanopore sensors have attracted attention as promising tools for electrochemical detection based on their ion current rectifying properties. This diode-like response, characterized by unequal current magnitude at equal but opposite applied potentials, is observed in nanopores with asymmetry in geometry, surface charge, and/or charge distribution.^1^ In conical-shaped glass nanopipettes, the charges on the internal surfaces attract counterions from the electrolyte solution, forming an electrical double layer (EDL) that, when comparable to the pore size, overlaps at the pore mouth and gives rise to perm-selectivity.^2−4^ Given the dependence of ICR on EDL overlap and, hence, nanopore surface charge, ion current rectifying nanopipettes can be employed for sensing applications by functionalizing the internal walls with analyte-specific probes, where analyte binding alters the surface charge and consequently modulates the ICR response.^5^

This sensing strategy has been widely adopted for detecting proteins,^6−8^ metal ions,^9−12^ and small organic molecules,^13−15^ leveraging the selectivity inherent to analyte-probe interactions to achieve subnanomolar detection limits. There are also limited examples of successfully integrating ion current rectifying nanopores or nanochannels to achieve sequence-specific and label-free nucleic acid detection. For instance, Fu et al. reported a sensitive and label-free sensing device for detecting DNA by modifying the surface of the nanopipette with cationic dendrimers, which electrostatically attach probe DNA onto the surface of the nanopipette. Sequence-specific DNA detection was realized by monitoring changes in ICR when hybridization occurs.^16^ Similarly, Ali et al. reported a peptide nucleic acid (PNA)-modified nanopore for selective and label-free detection of DNA.^17^ In 2016, Sun et al. designed a 3D nanochannel sensing device for the detection of DNA,^18^ with the biosensor displaying high specificity in the presence of interference and bovine serum. Liao et al. reported the development of a nanochannel biosensor utilizing phosphorodiamidate morpholino oligomers (PMO) as a capture probe for ultrasensitive and high sequence-specific detection of microRNAs.^19^ The biosensor demonstrated high specificity and sensitivity, with a detection limit of 1 fM in phosphate-buffer saline (PBS) and 10 fM in a serum sample. Despite these notable reports, using probe-functionalized nanopores for sensing clinically relevant nucleic acid targets remains scarce and experimentally challenging.

*Staphylococcus aureus* (*S. aureus*) is an opportunistic Gram-positive bacteria that can cause infections ranging from mild skin infections to life-threatening pneumonia and sepsis.^20−22^ Following the introduction of methicillin in the 1960s, *S. aureus* rapidly evolved, acquiring the *mecA* gene, which encodes the penicillin-binding protein 2a (PBP2a), leading to the emergence of methicillin-resistant *Staphylococcus aureus* (MRSA).^23,24^ The increasing prevalence of MRSA poses a serious global threat to public health due to its virulence and resistance to beta-lactam antibiotics and newer generations of antimicrobial compounds.^25−27^ MRSA detection commonly relies on classical bacterial culturing followed by antibiotic susceptibility testing to assess for antimicrobial resistance.^28−30^ While this methodology is simple and well-established, it is time and resource-intensive and prone to contamination.^31,32^ The “gold standard” for MRSA identification utilizes the polymerase chain reaction (PCR) to amplify a nucleic acid sequence inferring antibiotic resistance, such as within the *mecA* gene.^33,34^ This technique offers high sensitivity, selectivity, and rapid results compared to traditional culturing methods.^35−37^ However, the resulting amplified products must be analyzed using conventional gel electrophoresis or real-time quantitative PCR (qPCR) techniques. Gel electrophoresis visualizes PCR amplicons by staining them with toxic chemical dyes such as ethidium bromide or less-toxic proprietary alternatives (e.g., SYBR Safe), which intercalate with DNA duplexes allowing visualization of their separation based on size and charge. Although this method is widely used for its low cost, it is a complex process that lacks selectivity and can generate false positive signals as the dyes bind to any double-stranded DNA sequences.^38,39^ Alternatively, qPCR employs fluorescently labeled PCR primers or nucleotides, which are incorporated directly into the amplicon subsequent to the amplification reaction. qPCR, therefore, requires expensive equipment and reagents.^36^ Thus, despite the advantages of PCR, there is a need for simpler, cost-effective, and label-free alternatives (particularly those that possess miniaturization capabilities) capable of measuring the product of a DNA amplification reaction with high selectivity and sensitivity for rapid point-of-care diagnostics.

In this paper, we detail the development of an ICR-based biosensor (with a limit of detection as low as 350 fM) for detecting MRSA with a tunable dynamic range. Contrary to the traditional detection methods that often rely on fluorescent labeling and optical instrumentation for the readout, nanopipette-based sensors offer straightforward detection with unlabeled DNA and using simpler electronics, making the detection label-free, and cost-effective. Furthermore, the compact nature of the nanopipette setup and their low power requirements make them highly amenable for integration with portable devices allowing point-of-care detection. Herein, a probe sequence complementary to a region within the *mecA*-ending gene was selected and immobilized on the internal surface of a quartz nanopipette. Target detection was achieved by monitoring the change in rectification ratio following probe–target hybridization, including from a pathogen sample post-amplification without purification. To the best of our knowledge, DNA-functionalized nanopipettes have not been previously reported for pathogen detection from DNA isolated and amplified from a bacterial culture, nor has the ability to tune the sensitivity and dynamic range of ion-current rectifying sensors through the probe surface density been demonstrated. Our results are additionally supported by simulations with COMSOL Multiphysics, which explain how a lower surface probe loading can be used to achieve optimal sensor sensitivity. The developed sensor is a cost-effective, tunable, sensitive identification system, and when integrated with PCR, allows for label-free MRSA detection.

## Experimental Methods

### Materials and Reagents

All chemicals were purchased from Sigma-Aldrich unless otherwise stated below. (3-aminopropyl)triethoxysilane and 3-(maleimido)propionic acid *N*-hydroxysuccinimide ester were purchased from Fluorochem. Filamented quartz glass capillaries with 1 mm outer diameter and 0.7 mm internal diameter (#Q100–70–7.5) and microfil syringes were obtained from World Precision Instrument (WPI). Ag/AgCl electrodes were prepared in-house using 0.25 mm silver wires from Fisher Scientific. All solutions were prepared using Milli-Q- water from an Elga Purelab DV 35 water purification system. The Instagene matrix extraction kit was purchased from Bio-Rad Laboratories. All Primers were purchased from Eurofins. PCR reagents and Taq 2x master mix were purchased from NEB. All the oligonucleotides listed in Table S1 were purchased from Biomers.net GMbH.

### Nanopipette Fabrication and Characterization

Nanopipettes with a mean pore radius of 109 ± 20 nm were fabricated using a P-2000 laser pipette puller from Sutter Instruments with the following instrument-specific parameters: heat: 580, filament: 3, velocity: 55, delay: 128, pull: 110. The size of the nanopipettes was characterized by conductivity and scanning electron microscopy, as shown in the Supporting Information (Section S2).

### Functionalization of Nanopipette with Probe DNA

Probe strands of DNA were immobilized on the internal surface of nanopipettes via a three-step procedure. First, a solution of 5% (v/v) (3-aminopropyl)triethoxysilane (APTES) in ethanol was backfilled into the pipettes using a microfil syringe. The nanopipettes were left at room temperature for 1 h. The silane solution was removed, and the interior of the nanopipettes was rinsed thoroughly with ethanol to remove any loosely bound APTES molecules. The nanopipettes were then baked at 70 °C for 1 h. Next, the APTES-functionalized nanopipettes were filled with a solution of 1 mM 3-(maleimido)propionic acid *N*-hydroxysuccinimide ester (MPS) dissolved in a 1:9 (v/v) DMSO: 1X PBS overnight at room temperature, and then rinsed with 1X PBS to remove any unreacted MPS present in the nanopipettes. The maleimide-functionalization provides the platform to cross-link the amine-terminated silanes to the thiolated probe DNA strands. Finally, the nanopipettes were backfilled and incubated in a DNA probe solution for 4 h at room temperature. The nanopipettes were rinsed in 0.01 M KCl and were ready to use for sensing. Unless otherwise stated, cyclic voltammograms of each relevant modification step were collected in 0.01 M KCl before moving on to the next appropriate step.

### MRSA Detection via Probe-Functionalized Nanopipettes

Varied concentrations of the 15-mer target DNA were prepared in a 1X PBS solution. The probe-functionalized nanopipettes were backfilled and incubated with the target solution overnight at room temperature to ensure maximum hybridization. However, detection is achievable for an incubation time of 1 h or less, as discussed below for the clinically relevant samples. The nanopipettes were then thoroughly rinsed with 1X PBS to remove any unhybridized target.

### Electrochemical Measurements and Analysis

All *I–V* measurements were carried out using a 0.01 M KCl electrolyte at room temperature. Nanopipettes were backfilled with the electrolyte, and an Ag/AgCl wire working electrode was placed inside the pipette. The nanopipette was placed in a bulk electrolyte solution containing an Ag/AgCl wire reference electrode. The current was measured using a Biologic SP-200 potentiostat with an ultralow current at a high-speed option. The applied voltage was swept from −1 V to +1 V relative to the reference electrode at a scan rate of 0.1 V s^–1^. Measurements were performed with a filter bandwidth of 50 kHz. The noise was further filtered numerically using EC-Lab software (Biologic) by applying a moving average filter with a window size of 11 points. Data are reported as mean ± standard error of the mean, which is determined by dividing the standard deviation of the rectification ratio by the square root of the number of measurements (*n*). Each measurement represents a unique nanopipette sensor device.

### Pathogenic Sample Sensing

Type strains MRSA (ATCC43300), *Klebsiella pneumoniae* (*K. pneumoniae*) (ATCC23357), and methicillin-susceptible *S. aureus* (MSSA) (NCTC83254) were grown for 24 h on Trypticase Soy Agar. The genomic DNA of three colonies of each bacterium (of unknown CFU/mL) was extracted using the Instagene Matrix extraction kit (Bio-Rad Laboratories, Hercules, CA, USA). The primers used to amplify a 162 nucleotide region within the *mecA* gene of MRSA were based on published information from the European Union Reference Laboratory.^40^ Taq 2X PCR master Mix Master Mix (NEB, UK) was used to prepare master mix reactions for each target and loaded in 96-well. For all PCRs in this study, the denaturing temperature was set at 94 °C for 15 min, followed by 30 thermal cycles of 94 °C for 30 s. 59 °C for 60 s. and 72 °C for 60 s, followed by one cycle at 72 °C for 60 s and a final hold at 4 °C for 10 min. Following PCR amplification, the resulting products were analyzed by gel electrophoresis using a 2% [w/v] agarose gel in 0.5X Tris-borate-EDTA (TBE) buffer for 1.5 h, followed by imaging under UV light (Figure S8). The amplified product was diluted with 1X PBS at 1:100 μL. The probe-functionalized nanopipettes were dipped in the target solution for 1 h at room temperature and thoroughly rinsed with 1X PBS to remove any unhybridized target.

### Finite Element Simulations

A two-dimensional axisymmetric model incorporating the Poisson-Nernst–Planck and Navier–Stokes equations was utilized to model the developed sensor. The current rectifying model was constructed in COMSOL Multiphysics (version 6.2), with the Electrostatics and the Transport of Diluted Species modules used to incorporate the governing equations. Further information regarding these equations, the model geometry, meshing, and boundary conditions are provided in Supporting Information (Section S7).

## Results and Discussion

**Figure 1:**
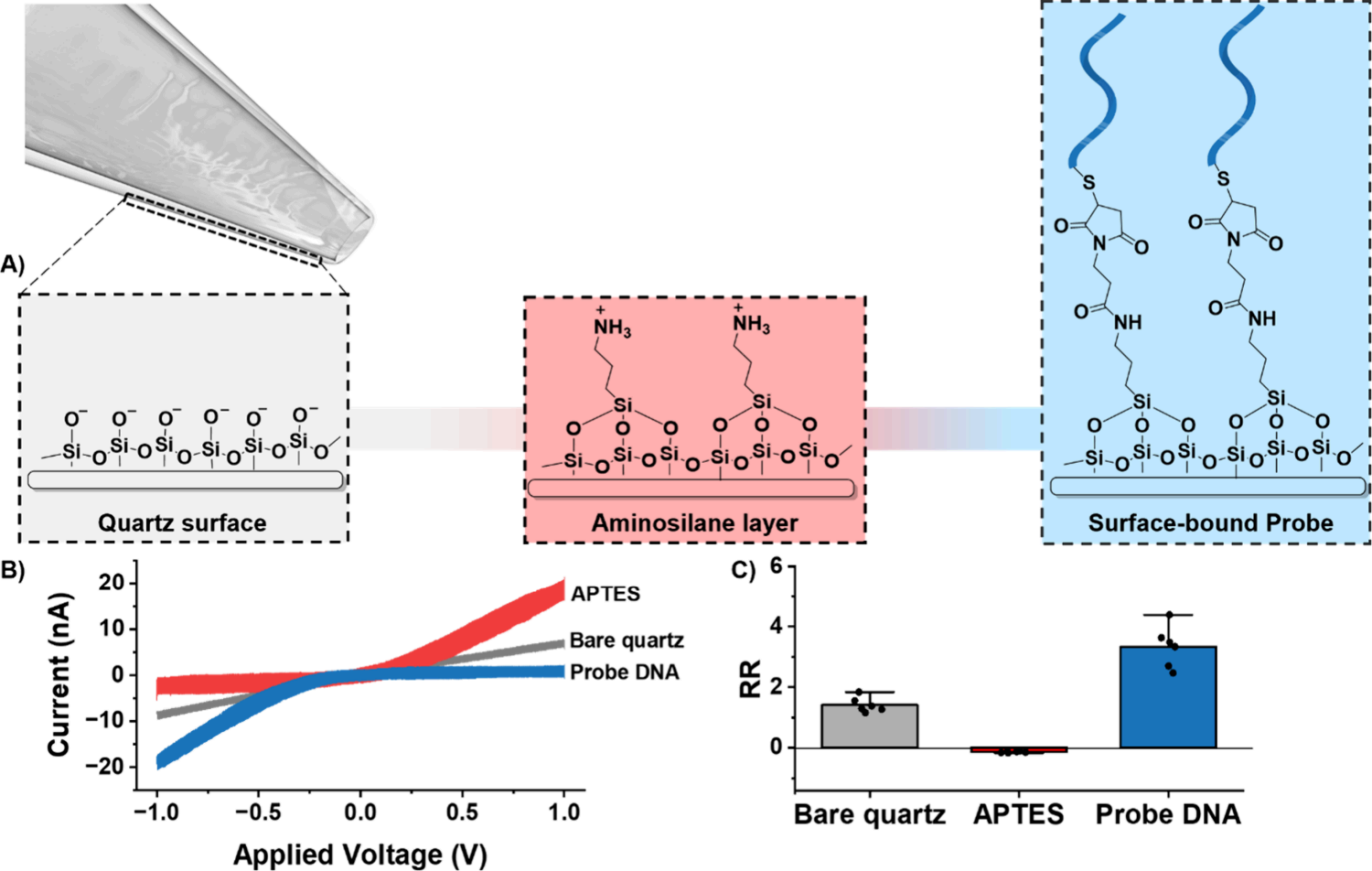
(A) Schematic of the stepwise functionalization of a quartz nanopipette surface. (B) Representative *I–V* curve for an unmodified (gray), APTES-functionalized, and probe DNA-modified (blue) nanopipettes. (C) The average rectification ratio for unmodified quartz (gray), APTES-modified (red), and probe-functionalized (blue) nanopipettes. Error bars are the standard error of the measurement of six unique nanopipettes.

It is well established that the changes to the internal surface of a nanopipette can significantly impact the mass and charge transport properties, which dictate the rectification observed. Consequently, the functionalization of glass nanopores can be assessed using *I–V* curves and the rectification ratio, RR, defined as the ratio of the observed current at equal but opposite polarities (eq 1).
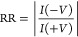
1Figure 1 shows the stepwise functionalization of the nanopore as a function of RR obtained from *I–V* curves. First, an *I–V* curve of the unmodified quartz nanopipette was obtained as a primary reference. At pH 7, the terminal silanol groups (SiOH, p*K*a 5.6) at the quartz surface dissociate, and the inner surface of the nanopipette becomes negatively charged, with an RR of 1.4 ± 0.1.^41^ Next, an amine-terminated silane was covalently attached to the internal surface of the nanopipette by treating its interior walls with 5% APTES in ethanol. The silanization process is initiated by the hydrolysis of reactive siloxanes, followed by the development of the covalent bond with the silanol groups on the surface of the nanopipette.^41^ Further, thermal curing promotes cross-linking between silanes on the surface of the nanopore.^42^ APTES functionalization rendered the nanopipette interfacial charge positive with an RR of 0.14 ± 0.01. The observed inversion in the direction of rectification is due to the protonation of the amine-terminated silanes (amine group, p*K*_a_ ∼ 9).^13^ Subsequently, a heterobifunctional maleimide cross-linker was attached as a precursor for the covalent attachment of the thiolated probe DNA to the nanopipette surface through thiol-maleimide click chemistry.^13^ As the phosphate backbone of DNA is negatively charged, the extent of rectification is similar to the initial cation selective state, with an RR of 3.3 ± 0.4.

### MRSA Target Detection

**Figure 2:**
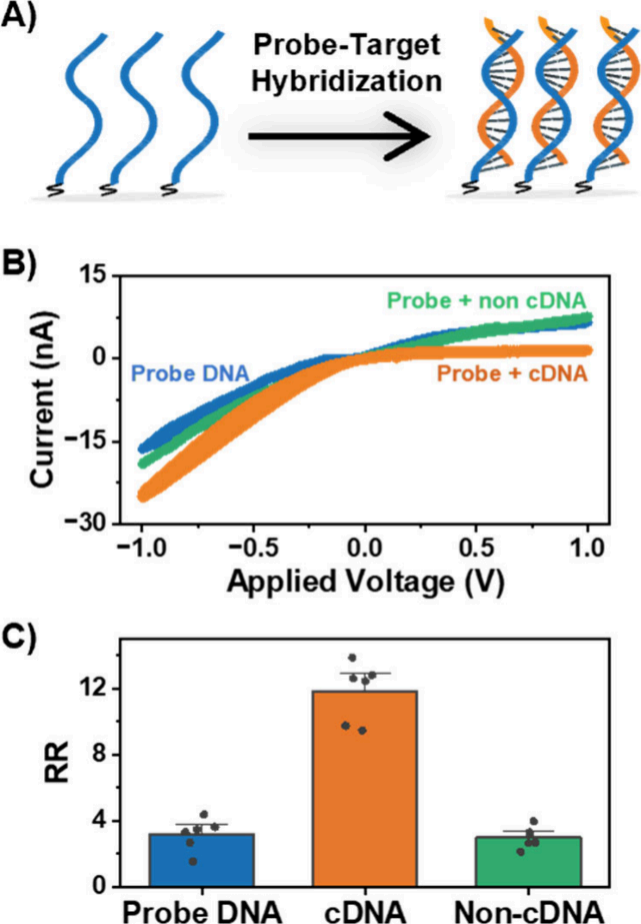
(A) Illustration of target detection via hybridization to complementary probe DNA functionalized on the nanopipette surface. (B) *I–V* curves and (C) The average rectification ratio bar chart for the probe-modified nanopipette (blue) after exposure to 10 μM of the complementary target DNA (orange) and noncomplementary DNA sequence (green). Error bars are the standard error of the measurement of six unique nanopipettes.

To develop nanopore sensors capable of detecting bacterial DNA indicative of MRSA, a model system was first developed, wherein the internal surface of the nanopipette is functionalized with a 15-mer DNA probe. This DNA sensor leverages the inherent specificity conferred by the Chargaff-specific base pairing to enable the detection of its complementary target sequence. The probe-functionalized nanopipettes were incubated overnight in an aqueous solution containing the complementary target sequence (initially 10 μM, chosen to ensure surface saturation). Following exposure to the target DNA, a significant increase in the rectification is observed from an RR of 3.3 ± 0.4 to 11.8 ± 0.7 (Figure 2C). This 3-fold increase in RR is attributed to the increased negative surface charge density upon the successful hybridization of the complementary target DNA with the probe DNA immobilized on the surface of the nanopipette. The response of probe-modified nanopipette to the presence of noncomplementary DNA sequence in high concentration conditions (also 10 μM) is also characterized to validate the specificity of target detection. A noncomplementary sequence was designed from the *bla*_KPC_ gene of *K. pneumonia* and modeled to ensure sufficient difference in secondary structure to the complementary *mecA* target sequence. When exposed to 10 μM of this noncomplementary sequence, an RR of 3.0 ± 0.3 is obtained (similar to the probe-only case), indicating that the probe-functionalized sensor selectively detects the *mecA* DNA of interest.

### The Dynamic Range and Limit of Detection Can Be Tuned through Variations in the Surface Probe Density

Numerous researchers have highlighted the importance of optimizing the surface density of probe molecules functionalized on the surface of gold and carbon-based electrodes.^43−45^ Notably, Paterson et al. showed that surface density influences the rate and efficiency of probe–target duplex formation on a gold electrode, with high surface densities displaying low binding efficiency and lower surface densities obtaining maximum probe–target hybridization due to a confluence of electrostatic interactions and probe conformation at the bulk interface.^46^ Similarly, Plaxco et al. demonstrated that varying the concentration of the probe aptamer during sensor fabrication leads to diverse probe surface density, offering a means to finetune binding efficacy and maximize the signal output obtained for target detection.^47^ It follows, then, that the number of probe strands per unit area on the nanopipette interior should also be crucial for ICR-sensing optimization. However, surface density cannot be directly quantified in nanopore sensors due to the nanoconfined environment, making surface density optimization more difficult than on bulk surface systems. Moreover, the nanoscale dimension of these sensors means there is an inherent increase in the exposed surface area of nanochannel for probe immobilization at the interface, thereby expanding the contact area between probe and target interaction alongside causing an increase in the local concentration of probe and target species as they are brought in proximity.^13^

**Figure 3:**
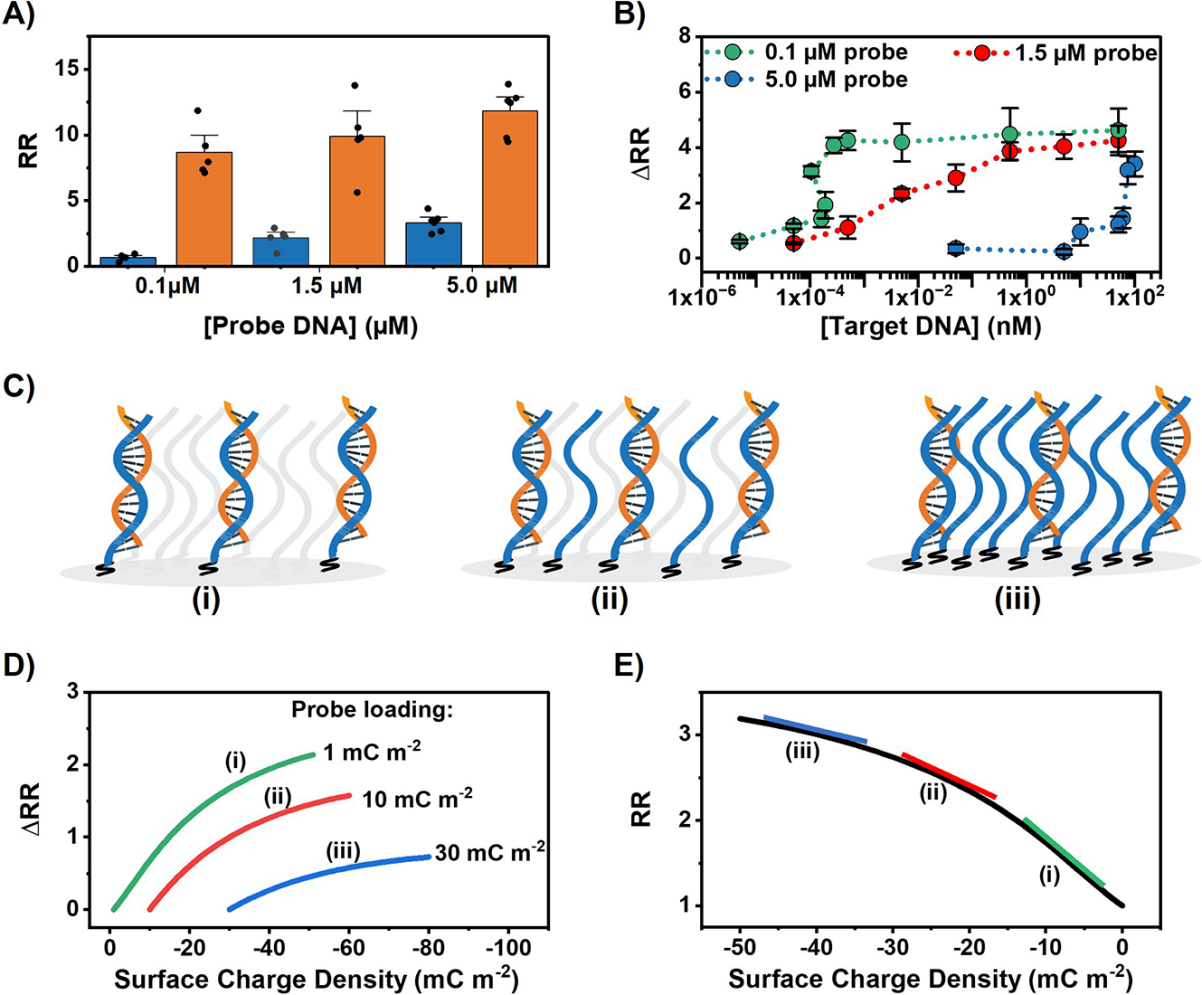
Ion-rectifying DNA sensors are most sensitive when the probe surface density is minimized. (A) The average RR of the nanopipettes functionalized with different probe loading before (blue) and after (orange) exposure to 10 μM target DNA. (B) The dose–response curves of nanopipettes functionalized with 0.1, 1.5, and 5 μM probe DNA. Error bars are the standard error of the measurement of five unique nanopipettes. (C) Illustration of the effect of varied probe loadings on target binding. (D) Finite element simulation of the change in RR when nanopipettes of different starting surface charge densities (probe loadings) undergo a change of surface charge density (analyte immobilization). (E) Finite element simulation of the RR of a nanopipette as a function of the surface charge density, with the gradient indicative of the sensitivity to variation of surface charge density, *d*(RR)/*d*(SC) (addition of infinite analyte), for a specified starting surface charge density (probe loading).

To investigate the influence of probe density, maleimide-modified nanopipettes were exposed to various probe DNA concentrations (0.1 μM, 1.5 μM, and 5.0 μM) during the immobilization step, with the objective of achieving different concentrations of probe loading on the nanopipette internal walls. As the concentration of probe DNA was decreased, a decrease in RR was observed (Figure 3). The highest RR was obtained for 5 μM at 3.3 ± 0.4, followed by 1.5 μM at 2.4 ± 0.5 and 0.1 μM at 0.7 ± 0.1 (Figure 3A). As the DNA probe density influences the surface charge density (because the phosphate backbone of DNA is negatively charged), the RR can be assumed to correlate with the probe loading. Thus, the observed trend in RR as a function of probe concentration exposure during the immobilization process demonstrates that the number of probe strands directly attached to the surface of the nanopipettes can be controlled by varying the probe concentration used during the functionalization process. This result provides valuable insight for tuning the properties of probe-functionalized nanopipettes for developing sensors that utilize ICR as the basis for sensing.

To assess the sensitivity of the system for target binding, nanopipettes with different probe loadings were exposed to target DNA at 10 μM, chosen to ensure surface saturation. Upon target hybridization, as anticipated, all probe loadings exhibited substantial increases in RR, attributable to the increased local anionic charge density following the probe–target hybridization reaction, with RR values of 11.8 ± 0.7, 9.9 ± 1.2, and 8.7 ± 0.8, respectively (Figure 3A).

Subsequently, the probe-functionalized nanopipettes were used to detect varying concentrations of target DNA and determine the limits of detection for each probe loading. The change in RR upon target binding was characterized by ΔRR (eq 2).
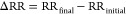
2

Regardless of the probe loading value, all functionalized nanopipettes exhibited a concentration dependent ICR response, with ΔRR proportional to the analyte concentration. Nanopipettes with different probe loadings also exhibited different dynamic ranges (Figure 3B). For example, when a probe concentration of 5 μM was used during nanopipette functionalization, the sensor displayed a dynamic range from 10 μM to 5 × 10^–2^ nM. Conversely, when a probe concentration of 0.1 μM was used during nanopipette functionalization, the dynamic range shifted, ranging from 10 μM to 5 × 10^–6^ nM. The limit of detection (LOD), defined as 3.3 times the standard deviation divided by the slope of the calibration curve, was estimated to be 350 fM, 360 pM, and 28 nM for nanopipettes functionalized with 0.1 μM, 1.5 μM, and 5.0 μM, respectively. Further details regarding the estimation of LOD for these experiments are provided in the Supporting Information. While these results are promising, the developed ICR sensors remain at this stage semiquantitative, and further optimization will be required to achieve full quantitative discrimination.

We propose that the lower detection limit (higher sensitivity) attained as the probe loading decreases is primarily attributed to the nanopipette being less sensitive to the modulation of surface charge that arises from target binding when it is initially highly charged. This supposition is supported by theoretical modeling using finite element analysis with COMSOL Multiphysics (Figure 3D, 3E). From the perspective of numerical modeling, both the probe loading and the analyte capture are purely an alteration of the nanopore surface charge density. As such, assuming that the magnitude of the initial surface charge of the nanopipette is proportional to the probe loading and the magnitude of the surface charge modulation from the initial surface charge density is proportional to the target concentration, sensitivity can be modeled with finite element simulation. As the DNA probe is negatively charged due to the negatively charged phosphate backbone, higher probe loadings correspond to a more negative initial surface charge density. Since the analyte is also negatively charged, the charge modulation will make the surface charge more negative. Figure 3D shows the expected ΔRR when nanopipettes of different starting surface charge densities (probe loadings) undergo a change of surface charge density (analyte capture). Our COMSOL model shows that nanopipettes with a lower probe density (initial surface charge) have a higher ΔRR response to any magnitude of analyte capture, supporting our experimental observations (Figure 3C). To further investigate the relationship between the sensitivity of a nanopipette to probe loadings, the rectification ratio and RR of the nanopipette were plotted as a function of surface charge density (Figure 3E). By considering the surface charge density (SC) as proportional to the probe loading, the first derivative of the curve at a specified surface charge represents the ΔRR in response to a change in surface charge (ΔSC). Counterintuitively, as shown in Figure 3E, the gradient of the tangent to the curve decreases as the surface charge density (probe loading) increases, which is why a smaller probe loading results in a higher response, as observed in the experimental data presented in Figures 3A and Figure 3B. To the best of our knowledge, the effect of surface density on modulating sensor sensitivity in nanopore systems has not previously been reported.

While we propose that the higher sensitivity of ion-rectifying nanopipette sensors at lower probe loading is primarily attributed to the nanopipette becoming less sensitive to the modulation of surface charge under these conditions, other factors at the molecular scale, commonly reported for sensor architectures at bulk interfaces, may also contribute as well. For example, lower probe loading will minimize unfavorable steric and electrostatic interactions, rendering the surface-bound probe more accessible for target binding.^48^

### Identification of MRSA from Bacterial Cultures via ICR

Encouraged by the demonstrated dose-responsive behavior observed for each probe surface density, we sought to explore the applicability of the sensor for label-free MRSA detection from laboratory-cultured samples. First, the nanopipettes were modified with 0.1 μM of an 81-mer probe sequence (Table S1). A longer probe sequence was used than in the investigations with synthetic targets to ensure the biosensor could selectively identify the DNA target diagnostic for MRSA in a clinically relevant matrix. We selectively amplified a 162-bp segment of the MRSA *mecA* gene, which included an 81-bp region complementary to the surface-bound probe sequence. 5 μL of the amplicons were analyzed by gel electrophoresis to ensure amplification was achieved (Figure S10), and the remaining was diluted in 1X PBS at a 1:100 ratio.

**Figure 4:**
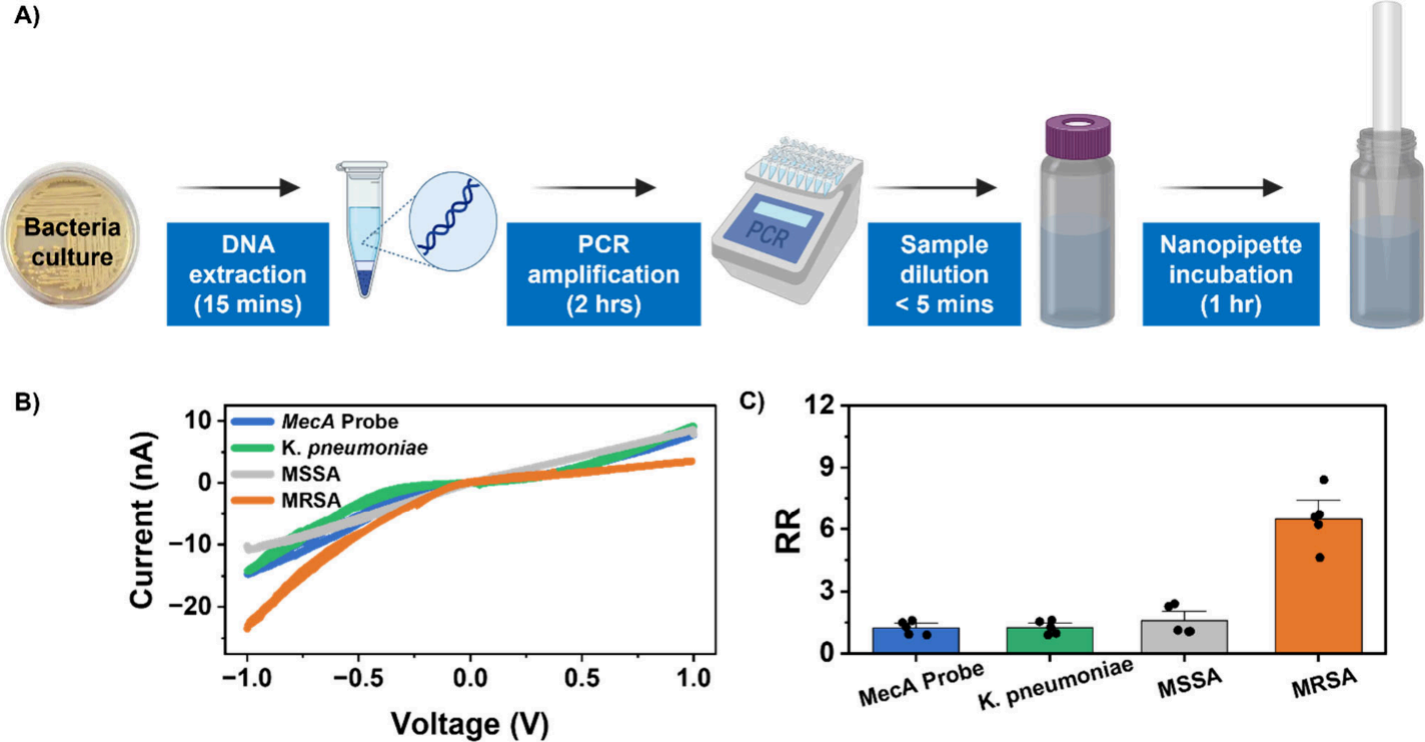
(A) Illustration of the sample preparation used for ICR-based DNA detection. (B) Sample *I–V* curves of a probe-functionalized nanopore before (blue) and after exposure to *K. pneumoniae* (green), MSSA (gray), and MRSA (orange). (C) A bar plot of the average rectification ratio of a probe functionalized nanopore before (blue) and after exposure to *K. pneumoniae* (green), MSSA (gray) and MRSA (orange). Error bars are the standard error of the measurement of five unique nanopipettes, each tested with a unique culture.

Following DNA extraction, the probe-functionalized nanopipettes were dipped in this solution for up to 1 h before recording their *I*-*V* response (Figure 4A). Initially, the probe-functionalized nanopore gave an RR of 1.2 ± 0.1. As expected, nanopipettes incubated in the MRSA-containing solution exhibited an increase in RR to 6.5 ± 0.5, indicating increased anionic charge on the surface of the nanopipette because of successful probe–target hybridization. (Figure 4B). Nanopipettes incubated in *K. pneumoniae* showed a minor RR change with an RR of 1.3 ± 0.1. To evaluate whether the developed sensing platform could differentiate MRSA and its closely related nontarget MSSA based on the presence of the *mecA* gene, an MSSA culture was also subjected to the same experimental procedure outlined above. Like the results obtained for *K. pneumoniae*, nanopipettes exposed to the MSSA solution displayed an average RR of 1.6 ± 0.3 (Figure 4C). Notably, in all tests, no sample purification step was carried out following PCR amplification, and the minimal changes observed from the *K. pneumoniae* and MSSA controls are likely due to some nonspecific binding of the primers, dNTPs, and Taq DNA polymerase in the PCR mixtures.

This system effectively differentiates bacterial cultures of antibiotic-resistant *S. aureus* from *K. pneumoniae* and antibiotic susceptible *S. aureus* based on the detection of DNA indicative of the *mecA* gene, post-amplification. Further testing will be required using clinical isolates and/or clinical specimens from infected patients, as well as a larger sample size to fully assess the sensor’s sensitivity, specificity and broad applicability in the clinical setting. Presently, we have demonstrated ICR sensing integrated with thermal amplification, and consequently, the requirement for trained personnel and a thermocycler remains similar to that of most routinely used molecular method, qPCR. However, ICR sensing utilizes unlabeled primers, and electronic measurements for detection, which can be performed with low-cost electronics that would be amenable to incorporation into a portable device. In particular, the cost-savings for unlabeled primers are significant, and with the nanopores themselves estimated at just 1 cent each, and portable potentiostats available for as little as a few hundred dollars, there is a significant cost-saving relative to qPCR. Additionally, we envisage that this ICR-based detection system should in future research be integrated with alternative isothermal amplification techniques, to eliminate the need for a thermocycler, reducing costs and increasing its capability as a point-of-care diagnostic test. Furthermore, ICR-based sensors should be utilized in tandem (rather than after) DNA amplification to achieve real-time sensing, removing the requirement for an incubation period.

**Figure 5:**
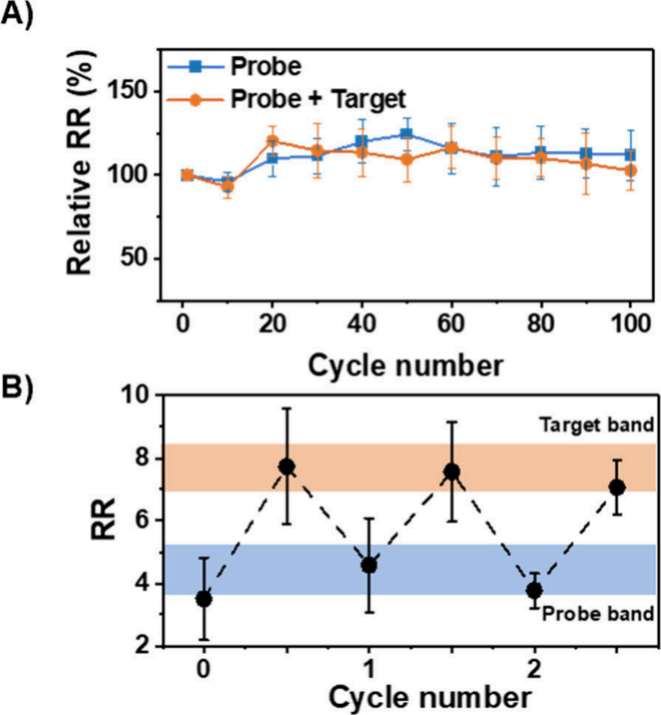
(A) Stability of the ICR response of a probe-functionalized nanopipette before and after target binding for 100 cycles. Each cycle is iterated in minute intervals, dividing the *x*-axis proportional to time (ca. 2 h). Error bars are the standard error of the measurement of three unique nanopipettes. (B) The reusability of the nanopore-based DNA sensor for up to two washes. Error bars are the standard error of five unique nanopipettes.

Finally, continuous voltammetric cycling and reusability studies were carried out to determine the stability of the probe-functionalized nanopipettes before and after target binding with clinically relevant samples. As shown in Figure 5A, the sensor response was stable with continuous interrogation for 100 cycles (ca. 2 h) with minimal hysteresis. Further, the reusability of the constructed sensor was investigated by stringent washing of the pipettes with hot deionized water (∼70 °C). As shown in Figure 5B, the rectification returned to a lower RR, corresponding to the regeneration of the initial probe-only state. The nanopipettes were then incubated in amplified target DNA overnight, regenerating the current–voltage response and rectification ratio associated with probe–target hybridization within the nanopore. This process was repeated for an additional two washes, indicating good reusability of the developed system.

## Conclusion

In this work, we demonstrate how DNA-functionalized nanopipettes can be used as ion-rectifying sensors for the selective and sensitive detection of DNA indicative of MRSA. Optimization studies were conducted to enhance the sensitivity of the nanopipette sensor by altering the probe DNA concentration at the nanoscale interface. The surface density on the nanopipette walls can be varied by controlling the probe DNA concentration utilized during functionalization, and subsequently, the dynamic range and sensitivity of the ion-rectifying sensor can be tuned. Finite element simulations using COMSOL Multiphysics reveal that an intrinsic property of ion-rectifying nanopipette sensors is that sensitivity to analyte binding is maximized when the initial surface charge density is low. At the lowest probe-loading investigated, we determined a detection limit of 350 fM. At this juncture, the sensor was integrated with the polymerase chain amplification reaction for low-cost, label-free MRSA detection without purification, and the reusability and stability of the nanopipette devices were also investigated. Future work will involve investigating the sensor response with clinical isolates, and determining the number of amplification cycles required for pathogen discrimination. The results presented here highlight the tunability and sensitivity of ion-current rectifying solid-state nanopipette sensors and their viability as a selective, cost-effective, and label-free detection platform for identifying antimicrobial infections.

## Supporting Information Available

Additional experimental data and technical details regarding the COMSOL model (PDF)

## Abbreviations

APTES: (3-aminopropyl)triethoxysilane
DNA: deoxyribonucleic acid
EDL: electrical double layer
ICR: ion current rectification
*I–V*: current–voltage
KCl: potassium chloride
MRSA: methicillin-resistant *Staphylococcus aureus*
PBP2a: penicillin-binding protein 2a
PBS: phosphate-buffered saline
PCR: polymerase chain reaction
PMO: phosphorodiamidate morpholino oligomer
PNA: peptide nucleic acid
qPCR: real-time quantitative polymerase chain reaction
RR: rectification ratio
*S. aureus*: *Staphylococcus aureus*
TBE: Tris-borate-EDTA

## References

[ref1] Wei C.; Bard A. J.; Feldberg S. W. Current Rectification at Quartz Nanopipet Electrodes. Anal. Chem. 1997, 69 (22), 4627–4633. 10.1021/ac970551g.

[ref2] Woermann D. Analysis of non-ohmic electrical current-voltage characteristic of membranes carrying a single track-etched conical pore. Nuclear Instruments and Methods in Physics Research Section B: Beam Interactions with Materials and Atoms 2002, 194 (4), 458–462. 10.1016/S0168-583X(02)00956-4.

[ref3] Siwy Z.; Fuliński A. A nanodevice for rectification and pumping ions. American Journal of Physics 2004, 72 (5), 567–574. 10.1119/1.1648328.

[ref4] Duleba D.; Dutta P.; Denuga S.; Johnson R. P. Effect of Electrolyte Concentration and Pore Size on Ion Current Rectification Inversion. ACS Measurement Science Au 2022, 2 (3), 271–277. 10.1021/acsmeasuresciau.1c00062. 35726254 PMC9204821

[ref5] Duleba D.; Johnson R. P. Sensing with ion current rectifying solid-state nanopores. Current Opinion in Electrochemistry 2022, 34, 100989 10.1016/j.coelec.2022.100989.

[ref6] Cai S.-L.; Cao S.-H.; Zheng Y.-B.; Zhao S.; Yang J.-L.; Li Y.-Q. Surface charge modulated aptasensor in a single glass conical nanopore. Biosens. Bioelectron. 2015, 71, 37–43. 10.1016/j.bios.2015.04.002. 25884732

[ref7] Ali M.; Yameen B.; Neumann R.; Ensinger W.; Knoll W.; Azzaroni O. Biosensing and Supramolecular Bioconjugation in Single Conical Polymer Nanochannels. Facile Incorporation of Biorecognition Elements into Nanoconfined Geometries. J. Am. Chem. Soc. 2008, 130 (48), 16351–16357. 10.1021/ja8071258. 19006302

[ref8] Ali M.; Schiedt B.; Neumann R.; Ensinger W. Biosensing with functionalized single asymmetric polymer nanochannels. Macromol. Biosci 2010, 10 (1), 28–32. 10.1002/mabi.200900198. 19685499

[ref9] Gao R.; Ying Y.-L.; Wu X.; Yan B.-Y.; Iqbal P.; Preece J. A. Ultrasensitive determination of mercury(II) using glass nanopores functionalized with macrocyclic dioxotetraamines. Microchimica Acta 2016, 183 (1), 491–495. 10.1007/s00604-015-1634-1.

[ref10] Liu X.; Liu C.; Yang J.; Zhang R.; Zeng Q.; Wang L. Detection and FEM studies of dichromate (Cr2O72-) by allyltriethoxysilane modified nanochannel. J. Electroanal. Chem. 2020, 858, 113818 10.1016/j.jelechem.2020.113818.

[ref11] Liu X.; Zeng Q.; Liu C.; Yang J.; Wang L. Experimental and Finite Element Method Studies for Femtomolar Cobalt Ions Detection by a DHI Modified Nanochannel. Analyst 2019, 144 (20), 6118–6127. 10.1039/C9AN01344J. 31532402

[ref12] Farrell E. B.; McNeill F.; Weiss A.; Duleba D.; Guiry P. J.; Johnson R. P. The Detection of Trace Metal Contaminants in Organic Products Using Ion Current Rectifying Quartz Nanopipettes. Anal. Chem. 2024, 96 (15), 6055–6064. 10.1021/acs.analchem.4c00634. 38569051 PMC11024892

[ref13] Nakatsuka N.; Faillétaz A.; Eggemann D.; Forró C.; Vörös J.; Momotenko D. Aptamer Conformational Change Enables Serotonin Biosensing with Nanopipettes. Anal. Chem. 2021, 93 (8), 4033–4041. 10.1021/acs.analchem.0c05038. 33596063

[ref14] Stuber A.; Douaki A.; Hengsteler J.; Buckingham D.; Momotenko D.; Garoli D.; Nakatsuka N. Aptamer Conformational Dynamics Modulate Neurotransmitter Sensing in Nanopores. ACS Nano 2023, 17 (19), 19168–19179. 10.1021/acsnano.3c05377. 37721359 PMC10569099

[ref15] Zhang S.; Chai H.; Cheng K.; Song L.; Chen W.; Yu L.; Lu Z.; Liu B.; Zhao Y.-D. Ultrasensitive and regenerable nanopore sensing based on target induced aptamer dissociation. Biosens. Bioelectron. 2020, 152, 112011 10.1016/j.bios.2020.112011. 32056734

[ref16] Fu Y.; Tokuhisa H.; Baker L. A. Nanopore DNA sensors based on dendrimer-modified nanopipettes. Chem. Commun. 2009, (32), 4877–4879. 10.1039/b910511e. 19652811

[ref17] Ali M.; Neumann R.; Ensinger W. Sequence-Specific Recognition of DNA Oligomer Using Peptide Nucleic Acid (PNA)-Modified Synthetic Ion Channels: PNA/DNA Hybridization in Nanoconfined Environment. ACS Nano 2010, 4 (12), 7267–7274. 10.1021/nn102119q. 21082785

[ref18] Sun Z.; Liao T.; Zhang Y.; Shu J.; Zhang H.; Zhang G. J. Biomimetic nanochannels based biosensor for ultrasensitive and label-free detection of nucleic acids. Biosens Bioelectron 2016, 86, 194–201. 10.1016/j.bios.2016.06.059. 27372572

[ref19] Liao T.; Li X.; Tong Q.; Zou K.; Zhang H.; Tang L.; Sun Z.; Zhang G.-J. Ultrasensitive Detection of MicroRNAs with Morpholino-Functionalized Nanochannel Biosensor. Anal. Chem. 2017, 89 (10), 5511–5518. 10.1021/acs.analchem.7b00487. 28429595

[ref20] Algammal A. M.; Hetta H. F.; Elkelish A.; Alkhalifah D. H. H.; Hozzein W. N.; Batiha G. E.; El Nahhas N.; Mabrok M. A. Methicillin-Resistant Staphylococcus aureus (MRSA): One Health Perspective Approach to the Bacterium Epidemiology, Virulence Factors, Antibiotic-Resistance, and Zoonotic Impact. Infect Drug Resist 2020, 13, 3255–3265. 10.2147/IDR.S272733. 33061472 PMC7519829

[ref21] Rayner C.; Munckhof W. J. Antibiotics currently used in the treatment of infections caused by Staphylococcus aureus. Internal Medicine Journal 2005, 35 (s2), s3–S16. 10.1111/j.1444-0903.2005.00976.x. 16271060

[ref22] Lakhundi S.; Zhang K. Methicillin-Resistant Staphylococcus aureus: Molecular Characterization, Evolution, and Epidemiology. Clin. Microbiol. Rev. 2018, 31 (4), e00020-18 10.1128/CMR.00020-18. 30209034 PMC6148192

[ref23] Vestergaard M.; Frees D.; Ingmer H. Antibiotic Resistance and the MRSA Problem. Microbiology Spectrum 2019, 10.1128/microbiolspec.GPP3-0057-2018. PMC11590431 30900543

[ref24] Kim C.; Milheiriço C.; Gardete S.; Holmes M. A.; Holden M. T. G.; de Lencastre H.; Tomasz A. Properties of a novel PBP2A protein homolog from Staphylococcus aureus strain LGA251 and its contribution to the β-lactam-resistant phenotype. J. Biol. Chem. 2012, 287 (44), 36854–36863. 10.1074/jbc.M112.395962. 22977239 PMC3481288

[ref25] Turner N. A.; Sharma-Kuinkel B. K.; Maskarinec S. A.; Eichenberger E. M.; Shah P. P.; Carugati M.; Holland T. L.; Fowler V. G. Methicillin-resistant Staphylococcus aureus: an overview of basic and clinical research. Nature Reviews Microbiology 2019, 17 (4), 203–218. 10.1038/s41579-018-0147-4. 30737488 PMC6939889

[ref26] Kaur D. C.; Chate S. S. Study of Antibiotic Resistance Pattern in Methicillin Resistant Staphylococcus Aureus with Special Reference to Newer Antibiotic. J. Glob Infect Dis 2015, 7 (2), 78–84. 10.4103/0974-777X.157245. 26069428 PMC4448330

[ref27] Wang X.; Chu C.; Deng Y.; Ma Y.; Yang M.; Luo H.; Huo D.; Hou C. Colorimetric and fluorescent dual-identification of Methicillin-Resistant Staphylococcus aureus (MRSA) using programmable CRISPR/Cas12a system. Microchemical Journal 2024, 197, 109873 10.1016/j.microc.2023.109873.

[ref28] Tille P. M. Bailey & Scott’s diagnostic microbiology. 15th ed.; Elsevier, 2022.

[ref29] CLSI M100: Performance Standards for Antimicrobial Susceptibility Testing, 30th ed.; Clinical and Laboratory Standards Institute, 2020. https://www.nih.org.pk/wp-content/uploads/2021/02/CLSI-2020.pdf (accessed 05-06-2024).

[ref30] van der Zwaluw K.; de Haan A.; Pluister G. N.; Bootsma H. J.; de Neeling A. J.; Schouls L. M. The Carbapenem Inactivation Method (CIM), a Simple and Low-Cost Alternative for the Carba NP Test to Assess Phenotypic Carbapenemase Activity in Gram-Negative Rods. PLoS One 2015, 10 (3), e0123690 10.1371/journal.pone.0123690. 25798828 PMC4370852

[ref31] Huletsky A.; Lebel P.; Picard F. J.; Bernier M.; Gagnon M.; Boucher N.; Bergeron M. G. Identification of methicillin-resistant Staphylococcus aureus carriage in less than 1 h during a hospital surveillance program. Clin Infect Dis 2005, 40 (7), 976–981. 10.1086/428579. 15824989

[ref32] Figdor D.; Gulabivala K. Survival against the odds: microbiology of root canals associated with post-treatment disease. Endodontic Topics 2008, 18 (1), 62–77. 10.1111/j.1601-1546.2011.00259.x.

[ref33] Taylor T. A.; Unakal C. G. Staphylococcus aureus infection; StatPearls Publishing, 2023. 28722898

[ref34] Al-Talib H.; Yean C. Y.; Al-Khateeb A.; Hasan H.; Ravichandran M. Rapid detection of methicillin-resistant Staphylococcus aureus by a newly developed dry reagent-based polymerase chain reaction assay. Journal of Microbiology, Immunology and Infection 2014, 47 (6), 484–490. 10.1016/j.jmii.2013.06.004. 23927820

[ref35] Velusamy V.; Arshak K.; Korostynska O.; Oliwa K.; Adley C. An overview of foodborne pathogen detection: In the perspective of biosensors. Biotechnology Advances 2010, 28 (2), 232–254. 10.1016/j.biotechadv.2009.12.004. 20006978

[ref36] Rajapaksha P.; Elbourne A.; Gangadoo S.; Brown R.; Cozzolino D.; Chapman J. A review of methods for the detection of pathogenic microorganisms. Analyst 2019, 144 (2), 396–411. 10.1039/C8AN01488D. 30468217

[ref37] Francois P.; Pittet D.; Bento M.; Pepey B.; Vaudaux P.; Lew D.; Schrenzel J. Rapid detection of methicillin-resistant Staphylococcus aureus directly from sterile or nonsterile clinical samples by a new molecular assay. J. Clin Microbiol 2003, 41 (1), 254–260. 10.1128/JCM.41.1.254-260.2003. 12517857 PMC149566

[ref38] Mouillesseaux K. P.; Klimpel K. R.; Dhar A. K. Improvement in the specificity and sensitivity of detection for the Taura syndrome virus and yellow head virus of penaeid shrimp by increasing the amplicon size in SYBR Green real-time RT-PCR. Journal of Virological Methods 2003, 111 (2), 121–127. 10.1016/S0166-0934(03)00167-8. 12880927

[ref39] Haines A. M.; Tobe S. S.; Kobus H. J.; Linacre A. Properties of nucleic acid staining dyes used in gel electrophoresis. ELECTROPHORESIS 2015, 36 (6), 941–944. 10.1002/elps.201400496. 25546455

[ref40] Stegger M.; Andersen P. S.; Kearns A.; Pichon B.; Holmes M. A.; Edwards G.; Laurent F.; Teale C.; Skov R.; Larsen A. R. Rapid detection, differentiation and typing of methicillin-resistant Staphylococcus aureus harbouring either mecA or the new mecA homologue mecALGA251. Clinical Microbiology and Infection 2012, 18 (4), 395–400. 10.1111/j.1469-0691.2011.03715.x. 22429460

[ref41] Duleba D.; Denuga S.; Johnson R. P. Reproducibility and stability of silane layers in nanoconfined electrochemical systems. Phys. Chem. Chem. Phys. 2024, 26 (21), 15452–15460. 10.1039/D4CP01181C. 38747528

[ref42] Jonkheijm P.; Weinrich D.; Schröder H.; Niemeyer C. M.; Waldmann H. Chemical strategies for generating protein biochips. Angew. Chem., Int. Ed. Engl. 2008, 47 (50), 9618–9647. 10.1002/anie.200801711. 19025742

[ref43] Steel A. B.; Herne T. M.; Tarlov M. J. Electrochemical Quantitation of DNA Immobilized on Gold. Anal. Chem. 1998, 70 (22), 4670–4677. 10.1021/ac980037q. 9844566

[ref44] Keighley S. D.; Li P.; Estrela P.; Migliorato P. Optimization of DNA immobilization on gold electrodes for label-free detection by electrochemical impedance spectroscopy. Biosens. Bioelectron. 2008, 23 (8), 1291–1297. 10.1016/j.bios.2007.11.012. 18178423

[ref45] Jalali Sarvestani M. R.; Madrakian T.; Afkhami A. Simultaneous electrochemical determination of Pb2+ and Cd2+ ions in food samples by a silver nanoparticle/COF composite modified glassy carbon electrode. Journal of Food Measurement and Characterization 2023, 17 (4), 3505–3514. 10.1007/s11694-023-01880-1.

[ref46] Peterson A. W.; Heaton R. J.; Georgiadis R. M. The effect of surface probe density on DNA hybridization. Nucleic Acids Res. 2001, 29 (24), 5163–5168. 10.1093/nar/29.24.5163. 11812850 PMC97561

[ref47] White R. J.; Phares N.; Lubin A. A.; Xiao Y.; Plaxco K. W. Optimization of Electrochemical Aptamer-Based Sensors via Optimization of Probe Packing Density and Surface Chemistry. Langmuir 2008, 24 (18), 10513–10518. 10.1021/la800801v. 18690727 PMC2674396

[ref48] Wong I. Y.; Melosh N. A. An electrostatic model for DNA surface hybridization. Biophys. J. 2010, 98 (12), 2954–2963. 10.1016/j.bpj.2010.03.017. 20550908 PMC2884251

