# Supplementary material for: Tuning Ion Current Rectifying Nanopipettes for Sensitive Detection of Methicillin-Resistant Staphylococcus aureus
Source: Anal Chem. 2025 Jan 21;97(4):2003–10. doi: 10.1021/acs.analchem.4c03510 (PMC11800174; doi:10.1021/acs.analchem.4c03510)
Supplement: Supplementary file 1 — ac4c03510_si_001.pdf [file ac4c03510_si_001.pdf]

Supporting Information:

Tuning Ion Current Rectifying Nanopipettes for Sensitive Detection of Methicillin-Resistant Staphylococcus Aureus

Shekemi Denuga,[1] Pallavi Dutta,[1] Dominik Duleba, [1] Guerrino Macori,[2,3] Séamus Fanning,[3,4] Robert P. Johnson.[1,3]*

1. School of Chemistry, University College Dublin, Belfield, Dublin D04 N2E5, Ireland.
2. School of Biology & Environmental & Biological Sciences, University College Dublin, Belfield, Dublin D04 N2E5, Ireland.
3. UCD-Centre for Food Safety, University College Dublin, Belfield, Dublin D04 N2E5, Ireland.
4. School of Public Health, Physiotherapy & Sports Science, University College Dublin, Belfield, Dublin D04 N2E5, Ireland.

*robert.johnson@ucd.ie

Table of Contents

S1. Nucleic Acid Sequences S2
S2. Nanopipette Characterization S3
S3.1 Verification of Surface Modification *via* ICR S4
S3.2 Verification of Surface Modification via Contact Angle Analysis S5
S4. Sample Current-voltage Curves for Varied Probe and Target Concentrations S6
S5. Limit of Detection and Limit of Quantification of the Sensor S7
S6. Finite Element Simulations........ S9
S7. PCR Amplification and Visualization via Gel Electrophoresis S11
S8. Stability of the Nanopore Sensor S12
References S13

S1. Nucleic Acid Sequences

Six oligonucleotide sequences (Table S1) were used in this investigation. The probe DNA sequences were designed with a thiol modification to enable binding to the maleimide group attached to the quartz surface.

Table S1: Nucleic acid sequences.

Sequence Name	5' Modification	Nucleic Acid Sequence (5' -> 3')	3' Modification	Length (bp)
15-mer Probe	/	TTC CAG GAA TGC AGA	Thiol C3	15
Complementary Target	/	TTC CAG GAA TGC AGA	/	15
K. pneumoniae Target	/	CGA AAC CGC TCG TAA ACA CA	/	20
MecA Probe	Thiol C6	TCC AGA TTA CAA CTT CAC CAG GTT CAA CTC AAA AAA TAT TAA CAG CAA TGA TTG GGT TAA ATA ACA AAA CAT TAG ACG ATA	/	81
Forward Primer	/	TCC AGA TTA CAA CTT CAC CAG G	/	22
Reverse Primer	/	CCA CTT CAT ATC TTG TAA CG	/	20

S2. Nanopipette Characterization

Nanopipette radii were determined using a previously established procedure in which the current-voltage (*I-V*) response is measured in a 0.1 M KCl electrolyte solution.[1] The inverse of the gradient of the slope was used to determine the resistance, *R*, from which the radius of the nanopipette (r) is calculated (Equation S1).

$$r = \frac{1}{\kappa R}\left(\frac{1}{\pi \tan\theta} + \frac{1}{4}\right) \qquad (Eq. S1)$$

Here, κ is electrolyte conductivity, and θ is the half-cone angle.[2] A sub-set of nanopipette radii was verified using scanning electron microscopy (SEM), as shown in Figure S1.

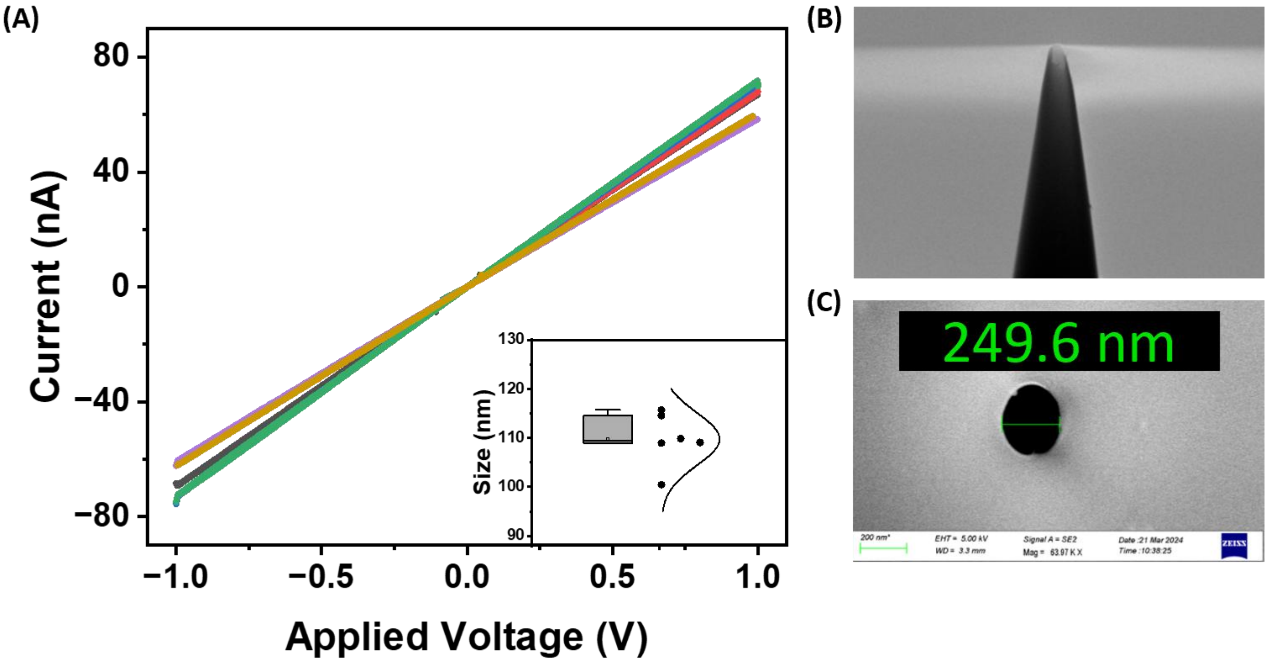

Figure S1: (A) Sample *I-V* responses for quartz nanopipette, with a mean radius of 109 ± 20 nm in 0.1 M KCl. (*n*=6). (B) Side view (C) End-on view SEM of the nanopipette with diameter indicated.

S3.1 Verification of Surface Modification *via* ICR

A series of control experiments were conducted to rigorously assess the relationship between the changes observed *via* ICR and the nanopipette surface modification by removing intermediate steps and recording the resulting *I-V* response (Figure S2). Initially, a bare quartz nanopipette, which is negatively charged, exhibited an average RR of 1.4 ± 0.1. The unmodified nanopipette was then exposed to the heterobifunctional maleimide cross-linker, followed by 5 µM of probe DNA (Control 1, Figure S2). In the absence of APTES, the addition of crosslinker and probe DNA did not significantly change the RR, which remained at 1.5 ± 0.3. Next, APTES-functionalized nanopipettes, with an RR of 0.14 ± 0.01, were exposed directly to 5 µM of probe DNA (Control 2, Figure S2). Without the maleimide cross-linker, there was a negligible increase in RR to 0.28 ± 0.01, attributed to nonspecific electrostatic interactions between the protonated amines of the surface of the nanopipette and the negatively charged DNA polymer. However, this indicates that the maleimide crosslinker is essential for the covalent immobilization of the probe DNA onto the nanopipette surface. Finally, unmodified nanopipettes were incubated in 10 µM complementary target DNA (Control 3, Figure S2). The resulting rectification remained similar to an unmodified nanopipette with an RR of 1.13 ± 0.03, indicating no non-specific binding between the desired target and the nanopipette surface. The data presented in Figure S2 confirm the success of the developed modification process for the nanopipette surface.

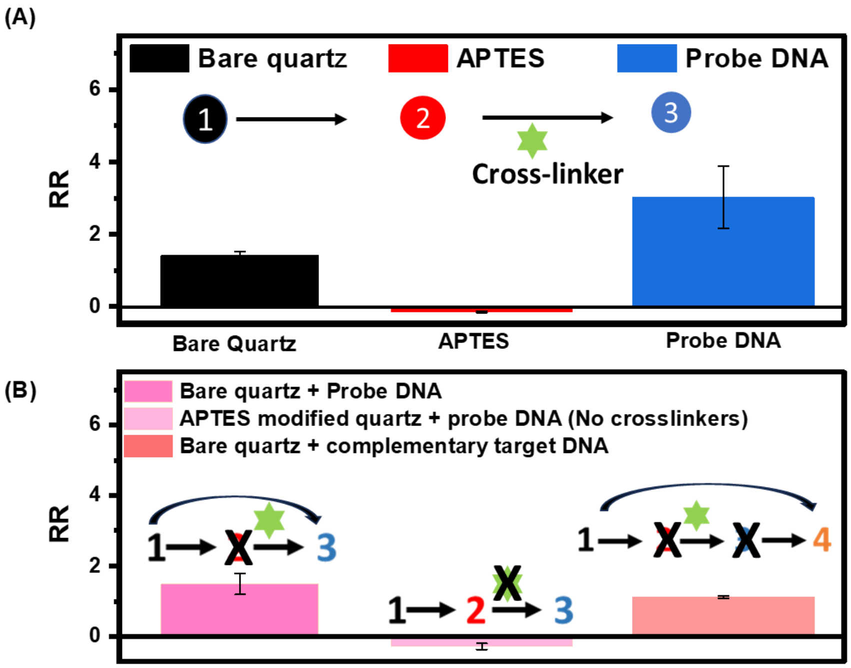

Figure S2: (A) The RR for the stepwise immobilization of probe DNA on the internal surface of quartz nanopipettes. (B) The RR, as an intermediary step of the modification process, was removed. *I-V* responses are obtained in 0.01 M KCl. Error bars are the standard error of the mean from a minimum of three unique nanopipette devices.

S3.2 Verification of Surface Modification via Contact Angle Analysis

Contact angle measurements were used to monitor the functionalization of a planar quartz wafer with the 15-mer probe DNA and for subsequent target detection (Figure S3) to validate the surface modification procedure. Static water contact angles of the functionalized surface were measured at room temperature using a sessile drop method with a 5 µl water droplet in a DSA30 (Krüss, Germany). As each step of the functionalization introduces additional functional groups on the surface, the modification of the quartz substrate could be monitored by measuring the water contact angle, with changes in the water contact angle related to the hydrophilicity or hydrophobicity of the surface. Upon silanization, the contact angle increased from 31.4 ± 0.6 to 62 ± 2. This increase in hydrophobicity confirmed the formation of a covalent siloxane bond between the quartz hydroxyl surface groups and APTES molecules containing alkane chains. Following the immobilization of the 16-mer probe and its complementary target DNA, the water contact angles decreased to 40 ± 5 and reached a minimum of 20 ± 2, respectively, indicating the increasing hydrophilic nature of the surface. This suggests the addition of highly polar groups on the quartz surface corresponding to the presence of DNA, a highly polar polymer, thereby confirming DNA functionalization and successful probe-target hybridization.

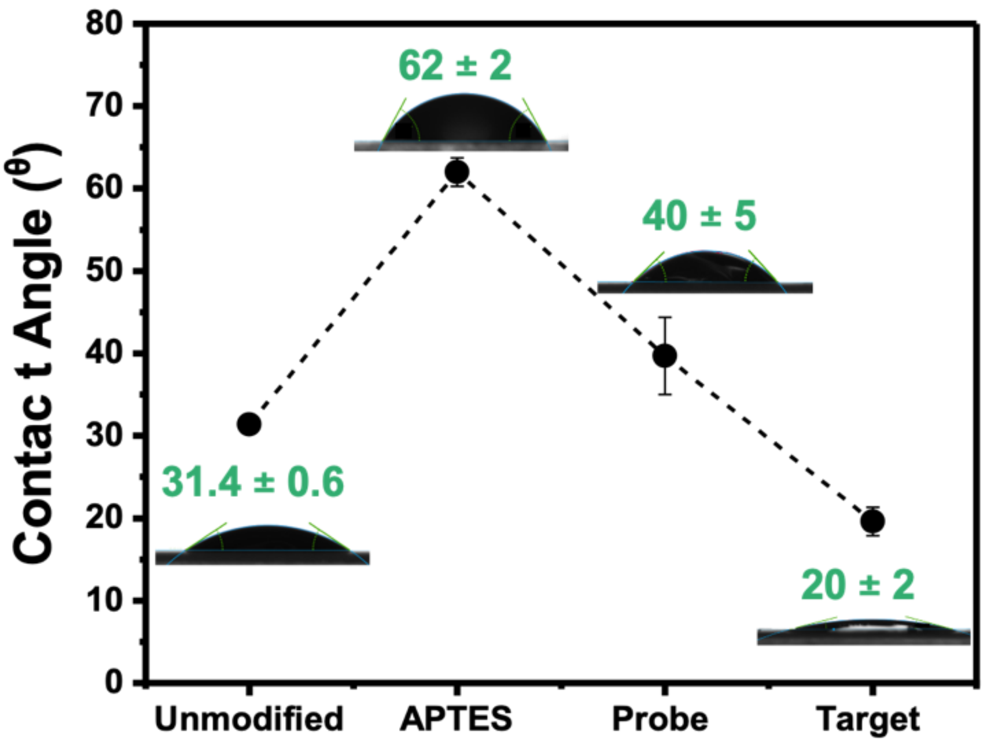

Figure S3: Contact angle measurements of the stepwise functionalization of a quartz wafer with probe DNA before and after target hybridization. Error bars are the standard error of the mean for six drops on the quartz slide.

S4. Sample Current-voltage Curves for Varied Probe and Target Concentrations

Maleimide-functionalized nanopipettes were incubated in 0.1, 1.5, or 5 µM probe DNA. Nanopipettes exposed to higher concentrations of probe DNA during surface functionalization subsequently displayed higher rectification, with the highest RR obtained for 5 µM at 3.3 ± 0.4, followed by 1.5 µM at 2.4 ± 0.5 and finally 0.1 µM at 0.7 ± 0.1 (Figure S4). This suggests that increasing probe concentration increases the number of probes tethered to the internal nanopipette surface. Hence, probe loading can be controlled by varying the concentration of probe DNA utilized in the functionalization process. As summarized in Figure 3 and Figure S4, decreasing the probe loading on the surface of the nanopipette enabled the detection of lower target concentrations.

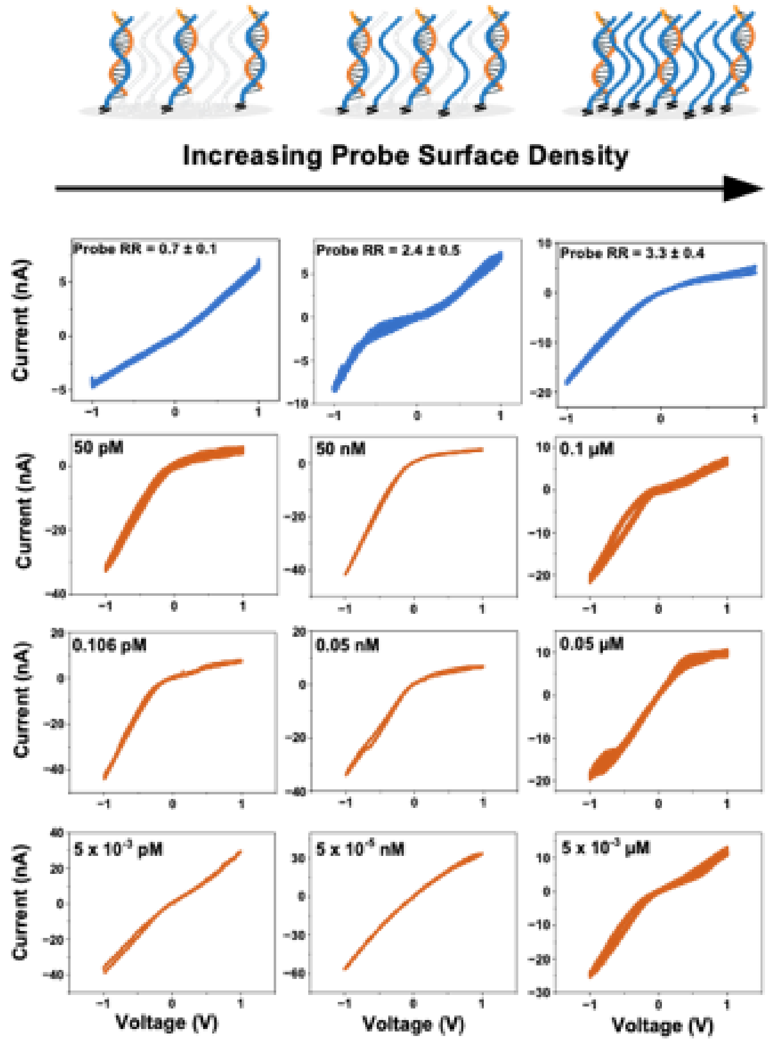

Figure S4: Representative *I-V* plots of probe functionalized nanopipettes before and after exposure to target DNA of varying concentrations.

S5. Limit of Detection and Limit of Quantification of the Sensor

Given the deviation from linearity obtained from the analyte concentration-dependent response data (Figure S5), we used two well-established methods to determine the most suitable approach for calculating the limit of detection (LOD) at all three probe loadings and report in the manuscript the method that gives the higher (less sensitive) limits of detection.

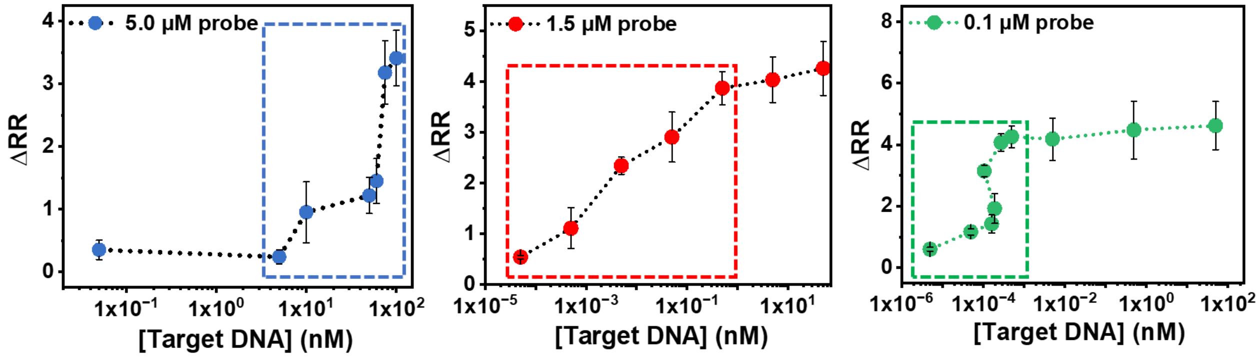

Figure S5: The limiting region of the dose-response curve of nanopipette-functionalized with 5, 1.5, and 0.1 μM probe DNA. Error bars are the standard error of the measurement of five unique nanopipettes.

Method 1: We identified a linear region of the calibration curve, then applied the commonly used analytical Equation S2 to determine the LOD, where $\bar{\sigma}$ is the standard deviation of the response, and S is the slope of the calibration curve in the linear region.

$$LOD = \frac{3.3\,\overline{\sigma}}{S} \qquad (Eq. S2)$$

Method 2: We determined the limit of the blank by assessing the mean signal in the absence of the analyte plus 1.645 times its standard deviation. Then, we calculated the LOD using Equation S3, which is defined as the blank signal plus 1.645 times the standard deviation of a sample with low concentration.[9]

$$LOD = Mean\ signal_{blank} + 1.645(\overline{\sigma}_{blank}) + 1.645(\overline{\sigma}_{low\ concentration\ samples}) \qquad (Eq. S3)$$

Using method 1, we estimated LODs of 350 fM, 360 pM, and 28 nM for nanopipettes functionalized with 0.1, 1.5, and 5 μM probe DNA, respectively. With method 2, we estimated LODs of 163 fM, 5 pM, and 70 nM, respectively. For this work, given the non-uniformity of the data across different probe loadings, we opted to report LOD based on method 1, as it is a more commonly used technique in analytical chemistry

and provides more conservative values. The limit of quantification (LOQ) for these sensors is further calculated using Equation S4, where $\bar{\sigma}$ is the standard deviation of the response, and S is the slope of the calibration curve in the linear region.

$$LOQ = \frac{10\,\bar{\sigma}}{S} \qquad (Eq. S4)$$

S6. Finite Element Simulations

Finite Element Analysis was carried out in the commercial software COMSOL® Multiphysics 6.2, where the Nernst-Plank equation (Equation S5) is solved self-consistently with the Poisson equation (Equation S6) to obtain the concentration and potential profiles:

$$\mathrm{J}_i = -\mathrm{D}_i \nabla c_i - \frac{z_i F}{RT} D_i c_i \nabla \Phi \qquad (Eq. S5)$$

$$\nabla^2 \Phi = -\frac{F}{\epsilon} \sum_i z_i c_i \qquad (Eq. S6)$$

where J_i denotes the ion flux, c_i the concentration, D_i the diffusion coefficient, and z_i the charge number of the ith, F is the Faraday constant, R is the ideal gas constant, T is the temperature, Φ is the electric potential and ϵ denotes the permittivity. A nanopore with a 100 nm pore radius and 10° internal half-cone angle is used in simulations. The pipette height extends to 40 µm while the bulk solution is 5 µm (Figure S6A), both sufficiently large to allow the concentration and potential gradients to naturally resolve back to boundary values. Boundary layer elements are used on the charged surfaces, and triangular elements are used elsewhere (Figure S6B). The model solves with 1519800 degrees of freedom (plus 80566 internal degrees of freedom).

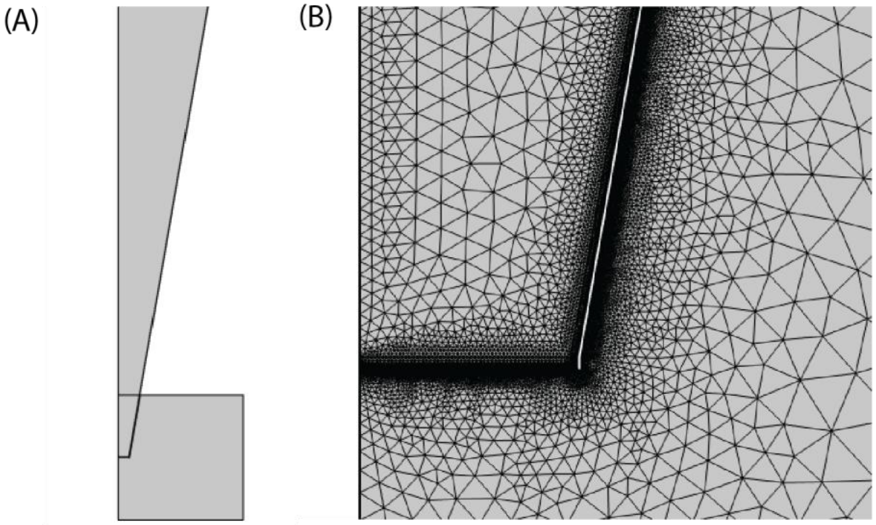

Figure S6: (A) The nanopipette geometry used for the model, and (B) a zoomed-in view of the meshed nanopipette tip region.

An average DNA density could be obtained from the literature and considering the number of phosphate backbones on the chain, this could be translated to a charge density per unit area. The main assumption that

this would introduce is that DNA density on bulk glass surfaces may differ greatly from the density obtained under nanoconfinement. Furthermore, as different parts of the nanoconfined surface experience very different electrostatic and ionic conditions (particularly at the regions where the potential drops and the regions where the ionic enrichment/depletion is the strongest), assuming that the nanoconfined surfaces have the same DNA density everywhere will also introduce errors. Lastly, the conformation of the DNA chain may also be important to account for. Although the volume charge density per DNA chain may be the same no matter what conformation the DNA takes, from the perspective of the electric double layer, a folded or flat-lying DNA chain may have effectively greater charge density on the surface, which would be very difficult to account for. Due to these difficulties, we went with the simpler representation to highlight the fundamental behavior of the system.

S7. PCR Amplification and Visualization via Gel Electrophoresis

Polymerase Chain Reaction (PCR) was performed using the ProFlex 96-well PCR system (ThermoFisher Scientific) to selectively amplify a 162-base pair (bp) segment within the MecA region. This defined region infers methicillin resistance in MRSA by encoding the penicillin-binding protein 2a (PBP2a). PCR was carried out in 25 μl reaction mixtures using a procedure previously reported.[3] The MRSA ATCC strain was amplified, along with *Klebsiella pneumoniae* and water as negative controls, to test the selectivity and specificity of the amplification process. Gel Electrophoresis was then carried out to verify the success of the PCR amplification. As shown in Figure S7, following the separation of the PCR samples through a 2% agarose gel matrix under UV light, a distinct band at the 162 bp position could be observed for the MRSA samples. In contrast, this band was absent for both negative controls, confirming that the target 162 bp sequence within the mecA region had been successfully amplified.

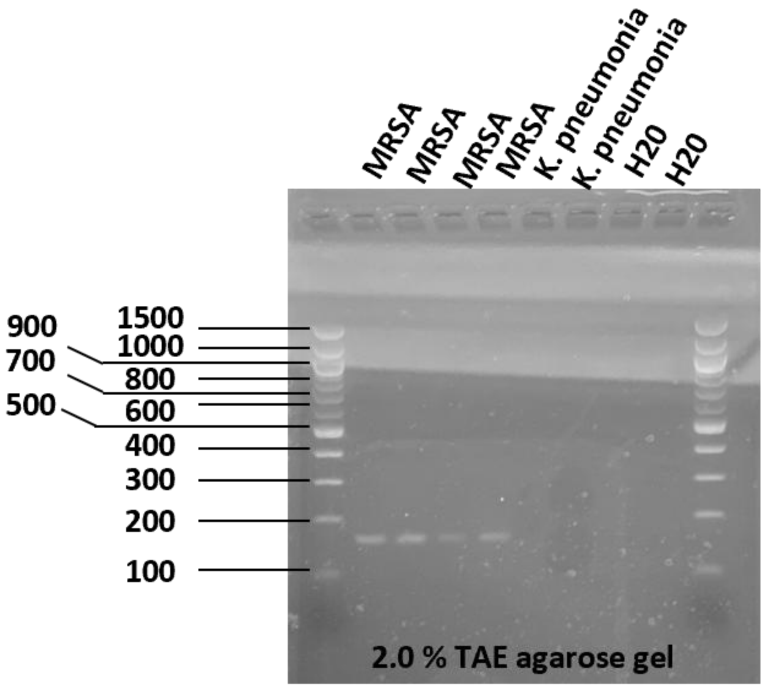

Figure S7: Visualization of selective amplification of MRSA.

S8. Stability of the Nanopore Sensor

The stability of the probe-modified nanopipettes was interrogated before and after target binding by subjecting the functionalized pipettes to continuous voltammetric cycling, as displayed in Figure S8. The RR observed for the probe-modified surface remained stable for up to 100 cycles (circa 2 h continuous measurement) with no considerable degradation in signal. After this, exposure of the probe-modified nanopipette to target DNA enhanced the negative rectification as expected, confirming the successful retention and fidelity of the probe and successful hybridization between the complementary sequences. The response to the probe-target modified pipette also remained stable for 100 cycles with minimal fluctuation, indicating the stability of the response (Figure S8).

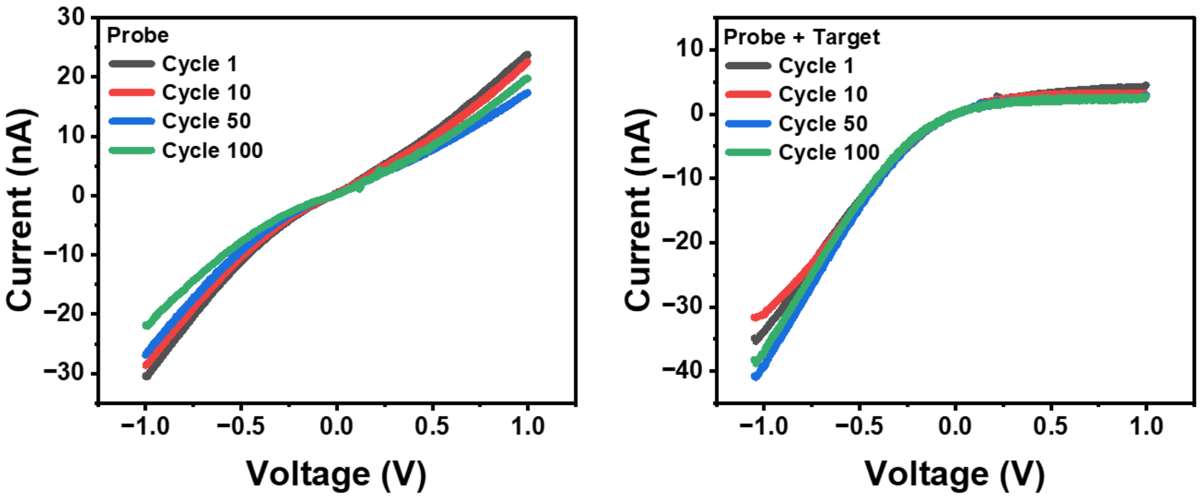

Figure S8: Representative *I-V* plots for nanopipettes (A) before and (B) after target binding as a function of continuous voltammetric cycling in 0.01 M KCl.

References

(1) Farrell, E. B.; Duleba, D.; Johnson, R. P. Aprotic Solvent Accumulation Amplifies Ion Current Rectification in Conical Nanopores. *The Journal of Physical Chemistry B* **2022**, *126* (30), 5689-5694. DOI: 10.1021/acs.jpcb.2c03172.

(2) Zhang, S.; Li, M.; Su, B.; Shao, Y. Fabrication and Use of Nanopipettes in Chemical Analysis. *Annual Review of Analytical Chemistry* **2018**, *11* (1), 265-286. DOI: 10.1146/annurev-anchem-061417-125840.

(3) Stegger, M.; Andersen, P. S.; Kearns, A.; Pichon, B.; Holmes, M. A.; Edwards, G.; Laurent, F.; Teale, C.; Skov, R.; Larsen, A. R. Rapid detection, differentiation and typing of methicillin-resistant Staphylococcus aureus harbouring either mecA or the new mecA homologue mecALGA251. *Clinical Microbiology and Infection* **2012**, *18* (4), 395-400. DOI: 10.1111/j.1469-0691.2011.03715.x.
